# Supplementary figures and images for: FhaA plays a key role in mycobacterial polar elongation and asymmetric growth
Source: mBio. 2025 Jan 21;16(3):e02526-24. doi: 10.1128/mbio.02526-24 (PMC11898655; doi:10.1128/mbio.02526-24)

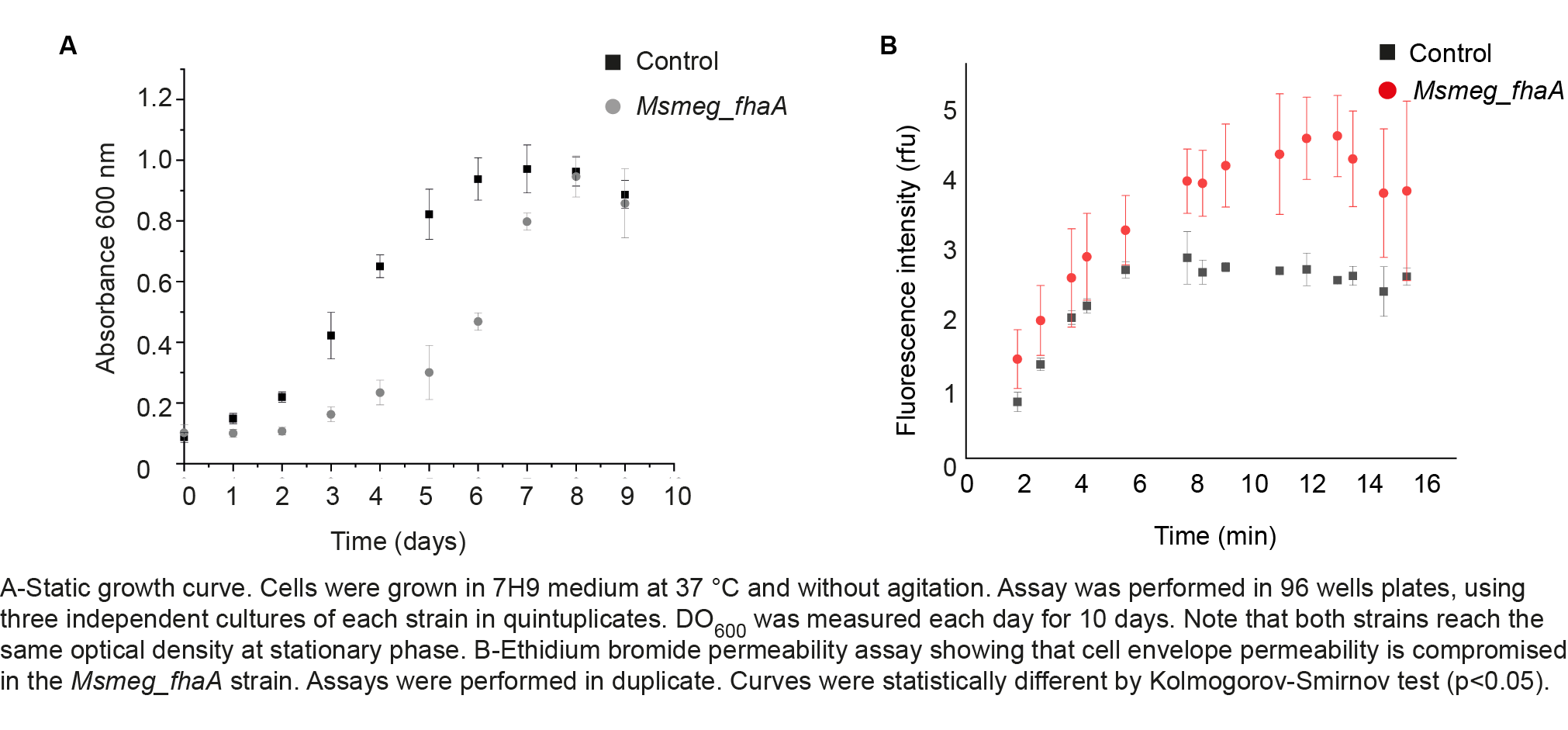

Supplement: Figure S1 — Static growth curve and permeability assay of Msmeg_fhaA and control strains. [file mbio.02526-24-s0001.tif]

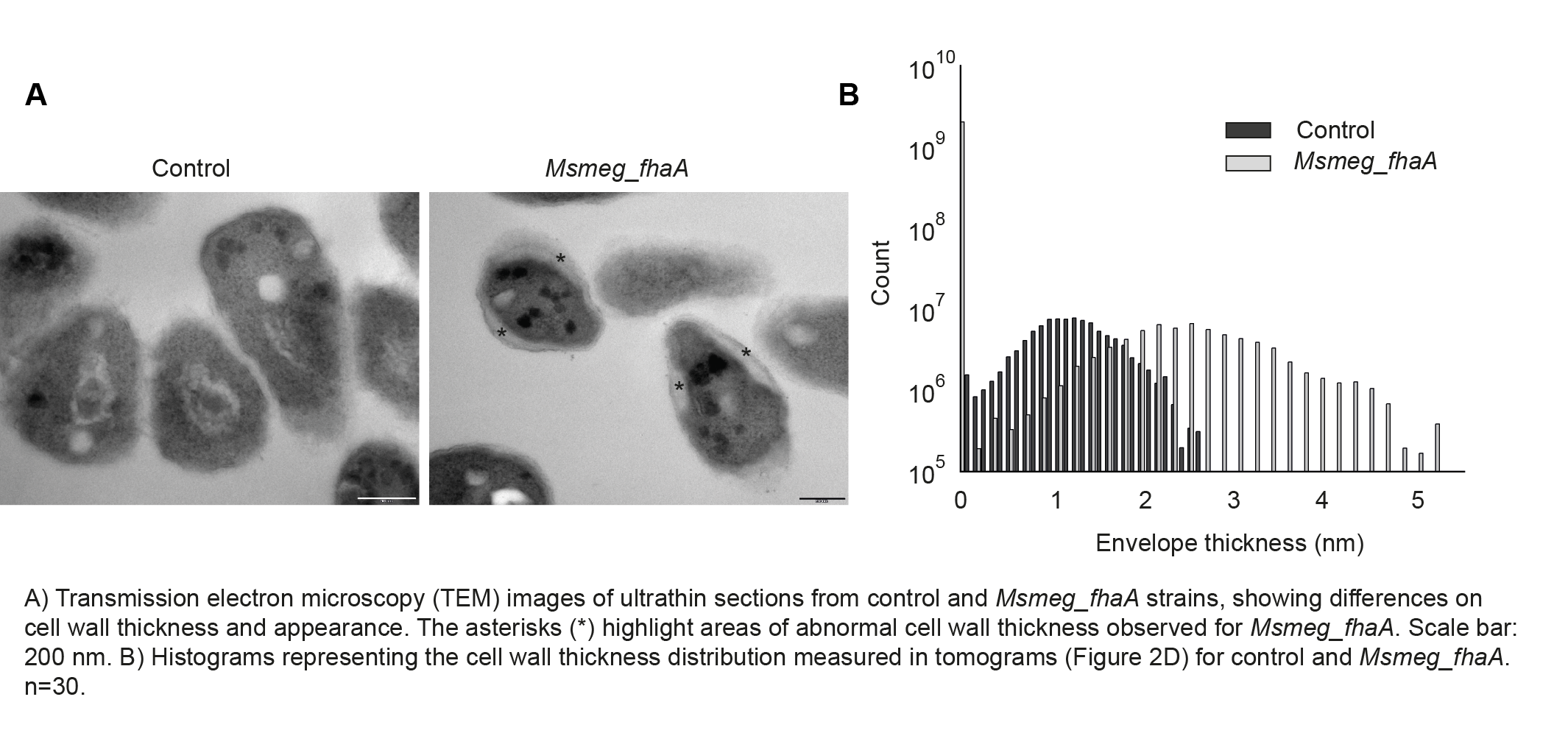

Supplement: Figure S2 — TEM images showing differences on cell wall thickness between control and Msmeg_fhaA strains. [file mbio.02526-24-s0002.tif]

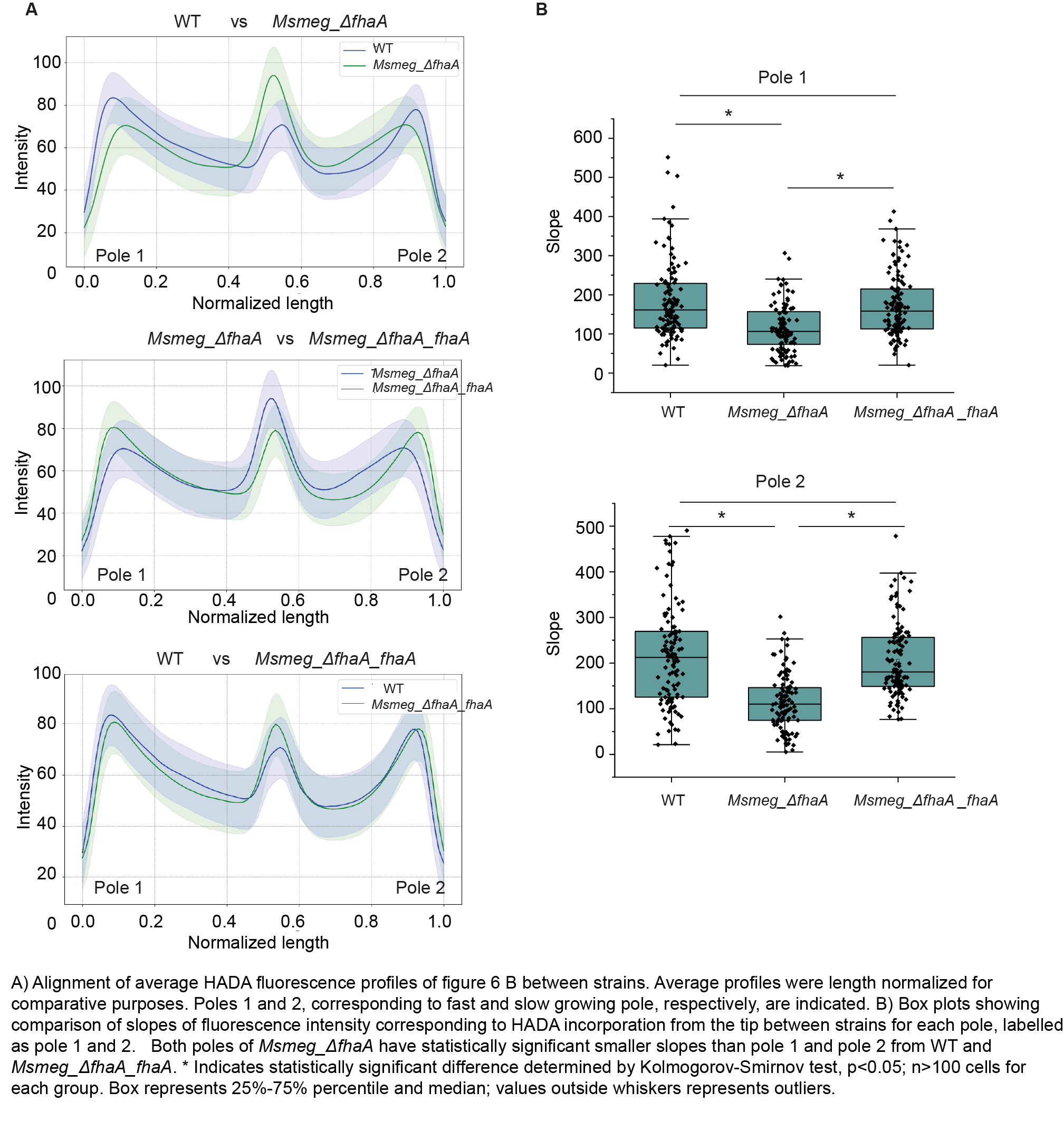

Supplement: Figure S3 — Comparison of HADA intensity profiles and HADA incorporation at the different poles for WT, Msmeg_ΔfhaA, and Msmeg_ΔfhaA_fhaA strains. [file mbio.02526-24-s0003.tif]
