# Supplementary material for: FhaA plays a key role in mycobacterial polar elongation and asymmetric growth
Source: mBio. 2025 Jan 21;16(3):e02526-24. doi: 10.1128/mbio.02526-24 (PMC11898655; doi:10.1128/mbio.02526-24)
Supplement: Legend — Video S1 legend. [file mbio.02526-24-s0004.docx]

**Supplementary video legend.**

3D reconstruction from virtual sections of a cell tomogram of a *Msmeg_fhaA* with an altered cell topography at the cell poles. There is an enlargement of the layer between the mycomembrane and inner membrane when compared to control strain. White layer represents the inner membrane; blue layer indicates peptidoglycan/arabinogalactan; light yellow indicates the mycomembrane.
